# Assessment of cardiac function in children with congenital adrenal hyperplasia: a case control study in Cameroon

**DOI:** 10.1186/s12887-017-0862-4

**Published:** 2017-04-20

**Authors:** J . Tony Nengom, S. Sap Ngo Um, D. Chelo, R. Mbono Betoko, J. Boombhi, F. Mouafo Tambo, A. Chiabi, S. Kingue, P. Koki Ndombo

**Affiliations:** 1 Faculty of Medicine and Biomedical Sciences of Yaounde I University, P.O Box: 14855, Yaounde, Cameroon; 2 Mother and Child Centre of the Chantal Biya Foundation, Yaounde, Cameroon; 3 Yaounde General Hospital, Yaounde, Cameroon; 4 Yaounde Gyneco Obstetric and Pediatric Hospital, Yaounde, Cameroon

**Keywords:** Cardiac function, Congenital adrenal hyperplasia, Children

## Abstract

**Background:**

High level of androgens found in congenital adrenal hyperplasia (CAH) seems to have a deleterious effect on heart function. We therefore evaluate cardiac function of children with CAH in comparison with a healthy group.

**Methods:**

We carried out a case-control study in the single endocrinology unit of the Mother and Child Center of Chantal Biya’s Foundation. Cases were matched for age and genotypic sex to 2 healthy controls. We analyzed the ejection fraction (LVEF), fractional shortening and left ventricular mass; output and cardiac index; E and A waves velocities, E/A ratio and the mitral deceleration time and diameter of the left atrium; tricuspid annular plane systolic excursion and pulmonary artery systolic pressure were also measured.

**Results:**

We included 19 patients with a median age of 6.26 ± 3.75 years and 38 controls stackable distribution. The left ventricular mass of cases was greater than that of controls. A case of reversible cardiomyopathy on hormone replacement therapy was found.

For the cases, the average ejection fraction was 71.95 ± 7.88%; the average fractional shortening was 40.67 ± 7.02%. All these values ​​were higher than those of controls, although the difference was not statistically significant. Diastolic left ventricular function was more impaired among the cases.

Right ventricular function was similar in both groups. These abnormalities were highly correlated to the late age at diagnosis and duration of treatment.

**Conclusion:**

This study shows an altered cardiac function in CAH compared to healthy control and highlights importance of an early diagnosis of cases, a tight control of androgens levels and a regular monitoring of cardiac function.

## Background

Children with Congenital Adrenal Hyperplasia (CAH), are exposed to high levels of androgens [1] leading to virilization of affected fetus. Besides their control of sexual function, androgens have multiple physiological functions including a major cardiovascular role [2, 3]. There is increasing evidence that patients with classical CAH have multiple vascular risk factors and that they are at increased risk for cardiovascular disease in adulthood [4–6]. Monitored measurements show that these patients also manifest elevated 24-h ambulatory blood pressure which can even lead to heart failure [7–9]. Various studies in children with CAH, show ventricular hypertrophy and impaired left ventricular function precisely the diastolic one by significant prolongation of both iso volumetric relaxation time and mitral deceleration time [9–11]. In Cameroon, there is very little data regarding the cardiovascular status and cardiac function of children followed up for CAH. This justify the present study with the aim of assessing cardiac function in affected children in Cameroon and compare them to a group of healthy children.

## Methods

This was a matched case-control study, performed over a 9 months’ period (October 2015 to July 2016). We included all recorded children affected with CAH in the national registry and followed in the single service of pediatric endocrinology of the country: the Mother and Child Center of the Chantal Biya Foundation (MCC/CBF). Cases aged 10 months to 15 years were matched for age and genotypic sex to 2 healthy controls. We used paired and unbalanced sampling (1 case per 2 controls) to increase the potency of the results and to limit the bias related to the sample size. The controls were recruited in children coming for a routine visit or vaccination. We excluded controls with another chronic condition, a known cardiovascular disease or acute illness modifying cardiac function (anemia, severe infection).

After an informed consent obtained from parents and assent from children aged >12 years, diagnosis circumstances, morphology (pelvic ultrasound, bone age) and lab results (17 hydroxyprogesterone, karyotype and biomolecular diagnosis when available) at diagnosis were noted. Height and weight were measured to the nearest 0.1 cm and 0.1 kg. Three blood pressures were measured and the mean noted for each case and control, with an electronic sphygmomanometer (Omron HEM 712C, Kyoto, Japan). The blood pressure was derived in percentile for height and genotypic sex. A general cardiac examination was done by a senior cardiologist.

Thereafter, a standard echocardiography in time mode motion, two-dimensional and Doppler [12, 13] was performed in both cases and controls with Siemens Acusson Cypress® (Siemens, Munich Germany) ultrasound machine.

The variables analyzed were:

- For the left ventricle: ejection fraction, fractional shortening, stroke volume and left ventricular mass; cardiac output and index; the E and A waves velocities, the E/A wave ratio and the mitral deceleration time;
- For the right ventricle: tricuspid annular plane systolic excursion (TAPSE). The pulmonary artery systolic pressure was also measured.

Height^2.7^ (in meters) has been validated as an indicator of lean body mass and has been recommended for indexing LVM. Use of height^2.7^ to index LVM also minimizes the effect of age, gender and race [14–17]. Stroke volume was indexed by the body surface area [18].

Statistical analyses were done using Microsoft® Excel 2013 and SPSS 20 software from IBM. Average and proportions served to describe quantitative and qualitative data. Comparison was done with the T test of Student (for normally distributed variable and homogeneity of variances) and a suitable non-parametric test Kruskall Wallis. Multivariate analysis was done to evaluate correlation between variables (Spearman). A *p* value <0.05 was statistically significant.

The study received ethical approval from institutional board review of Faculty of medicine and biomedical sciences of Yaounde.

## Results

We included 19 cases and 38 controls aged 10 months to 15 years with an average age of 6.26 ± 3.75 years. The most represented age was 4 years, with a proportion of 21% of cases. Among cases, 5 were boys and 14 girls. After the karyotype, only one was actually genotypically male (XY). As diagnosis circumstances, abnormal genitalia was the most represented (84.2%).The average age at diagnosis was 3.7 years (1 month to 12 years) (Tables 1 and 2)*.*

**Table 1:** General characteristic of study population

Variable	Cases	Controls
N	19	38
Phenotype (girls/boys)	14/5	36/2
Genotype (XX/XY)	18/1	-
Age (years)	6 ± 4	6 ± 4
Weight (kg)	23.8 ± 13.4	23 ± 11.6
Height (cm)	113.3 ± 26.2	118.5 ± 24.2
BMI	17.1 ± 2.9	15.4 ± 2
Heart rate (beats/min)	94.9 ± 20.2	100 ± 15.6

*BMI* Body Mass Index

**Table 2:** Clinical, biomolecular presentation of cases

		Number	Percent
Diagnosis circumstances	Abnormal genitalia	16	84.2
	Absence of testis	2	10.5
	Breast development	1	5.3
Age at diagnosis Median(range)	3.7 years (1 month-12 years)	-	-
Advance bone age Y/N	Yes/No	19/0	100/0
Salt wasting syndrome	Yes/No	4/15	21.1/78.9
Type of enzyme deficiency	11 β Hydroxylase deficiency	15	78.9
	21 Hydroxylase deficiency	4	21.1

Advance bone age was found in all patients at diagnosis. The most enzyme deficiency found was that of 11- βOH (11 Beta-hydroxylase), 78.9% of cases. Achondroplasia was found in 2 cases and one patient present with dilated cardiomyopathy diagnosed at 1 year of life, reversible on hormone replacement therapy (Table 2).

**Fig. 1:**
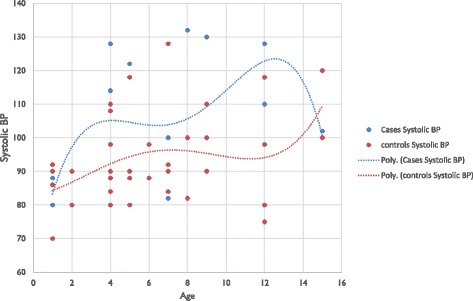
Systolic blood pressure of the study population

**Fig. 2:**
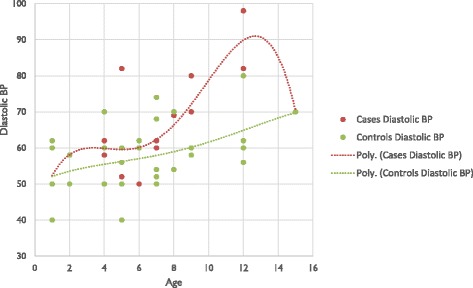
Diastolic blood pressure of the study population

Blood pressure was significantly higher in cases than controls, either systolic or diastolic (*p = 0.025*) (Figs. 1 and 2).

Concerning systolic function of the left ventricle, cases’ average ejection fraction (LVEF) was 71.95 ± 7.88%, VS 69.29 ± 7.95% in controls. The average fractional shortening (LVFS) was 40.67 ± 7.02% and 37.25 ± 5.64% for cases and controls respectively. In addition, mean stroke volume (SV) was 47.56 ± 13.58 ml/m^2^ and 43.15 ± 10.83 ml/m^2^ in cases and controls, respectively. The average cardiac output of cases was 3.76 ± 1.54 L/min. Mean cardiac index was 4.40 ± 1.07 L/min/m^2^ for cases and 4.38 ± 0.94 L/min/m^2^ for controls. Although cases values were above controls, the differences were not statistically significant (Table 3).

**Table 3:** Average comparisons of left ventricular ejection fraction, stroke volume, cardiac output, cardiac index, fractional shortening

	Cases	Controls	*P* value
LVEF % mean (range, SD)	71.95 (60–83, 7.8)	69.29 (51–92, 7.9)	0.238
SV ml/m^2^, mean (range, SD)	47.56 (20.1–72.6, 13.58)	43.15 (24.5–72.6, 10.83)	0.190
LVFS % mean (range, SD)	40.67 (31.10–51.16, 7.02)	37.25 (25.78–49.61, 5.64)	0.052
Cardiac output L/min, mean (range, SD)	3.376 (0.87–7.06, 1.54)	3.58 (1.55–5.88, 1.11)	0.620
Cardiac index, mean (range, SD) L/min/m^2^	4.40 (2.23–6.10, 1.07)	4.38 (2.63–6.55, 0.94)	0.939

*LEVF* Left Ventricular Ejection Fraction, *SV* Stroke Volume, *LVFS* left ventricular fractional shortening, *SD* Standard Deviation

Diastolic left ventricular function was more impaired among the cases. Thus, we found an average E wave velocity of 0.80 ± 0.32 m/s, lower than that of controls; an A wave velocity of 0.76 ± 0.35 m/s, higher than that of the controls, an average E/A ratio of 1.38 ± 0.96 less than the value of controls and a longer deceleration time, 147.58 ± 42.81(milliseconds) ms (Table 4).

**Table 4:** Average comparisons of E and A waves velocities, E/A ratio and mitral deceleration time

	Cases	Controls	*P* value
E wave velocity mean (range, SD) m/s	0.80 (0.33–1.27, 0.32)	0.99 (0.76–1.37, 0.13)	0.081
A wave velocity mean (range, SD) m/s	0.76 (0.36–1.53, 0.35)	0.57 (0.38–0.74, 0.10)	0.082
E/A RATIO mean (range, SD)	1.38 (0.37–3.53, 0.96)	1.80 (1.27–2.61, 0.33)	0.069
Mitral deceleration time ms mean (range, SD)	147.48 (78–228, 42.81)	137.47 (66–222, 40.3)	0.406

In terms of the systolic function of the right ventricle, there was no statistically significant difference between the systolic excursion of the plan of the tricuspid annulus (TAPSE) of cases and controls. In contrast, pulmonary pressures were within normal values while significantly higher in controls. However, we noted a case of CAH with a systolic pulmonary arterial hypertension (Table 5).

**Table 5:** Comparison of averages TAPSE and pulmonary artery systolic pressure between the two groups

	Cases	Controls	*P* value
TAPSE mean (range, SD) mm	19.86 (12.9–25.8, 3.44)	20.18 (1.9–32.2, 4.49)	0.785
Systolic pulmonary pressure mean (range, SD) m/s	13.16 (7–25, 4.68)	15.56 (8.5–24.5, 3.96)	0.047

*TAPSE* Tricuspid Annular Plane Systolic Excursion

The left ventricular mass index (LVMI) of cases was statistically greater than that of controls with an average value of 46.02 ± 17.14 g/m^2.7^. The end diastolic and systolic diameters of the left ventricle and the left atrium were almost identical in the two groups (Table 6).

**Table 6:** Comparison of left ventricle’s diastolic and systolic diameters, left atrium diameter and left ventricular mass

	Cases	Controls	*P* value
LVDd mean (range, SD) mm/m^2^	35.99 (19.4–47.3,7.9)	35.46 (24.1–47.1, 4.94)	0.755
LVSd mean (range, SD) mm/m^2^	21.31 (9.7–28, 4.90)	22.22 (13.8–32.2, 3.55)	0.427
LAD mean (range, SD) mm/m^2^	23.41 (13.6–32.7, 4.62)	22.71 (16.3–31.7, 3.64)	0.534
LVMI mean (range, SD) g/m^2.7^	46.02 (25–84.67, 17.14)	31.64 (20.2–46.9, 7.64)	**0.000**

*LVDd* Left Ventricular Diastolic diameter, *LVSd* Left Ventricular Systolic diameter, *LAD* Left Atrial Diameter, *LVMI* Left Ventricular Mass Index

The bold texts represent the result with a statistically significant differences

While analyzing correlation, we found that age at diagnosis does not influence the left ventricular mass index. A relationship was found between the diameter of the left atrium and age at diagnosis. For the diameter of the aorta, the correlation was very strong with age at diagnosis (*p* = 0.00) (Table 7). The duration of treatment does not influence the LVEF and LVFS (Table 8).

**Table 7:** Correlation between cardiac function parameters and age at diagnosis

Parameters	correlation coefficient (Spearman)	*P* value
LVEF	-0.1	0.685
SV	0.382	0.106
LVFS	0.004	0.988
LVMI	0.148	0.544
Cardiac output	0.441	0.059
Cardiac index	0.202	0.408
E/A Ratio	0.247	0.307
E wave velocity	0.191	0.434
A wave velocity	−0.257	0.289
Mitral deceleration time	0.371	0.118
LAD	**0.492**	**0.033**
TAPSE	0.447	0.055
Systolic pulmonary pressure	−0.057	0.817
Aortic diameter	**0.825**	**0.000**

*LEVF* Left Ventricular Ejection Fraction, *SV* Stroke Volume, *LVFS* Left Ventricular Fractional Shortening, *LVMI* Left Ventricular Mass Index, *LAD* Left Atrial Diameter, *TAPSE* Tricuspid Annular Plane Systolic Excursion

The bold texts represent the result with a statistically significant differences

**Table 8:** Correlation between the parameters of cardiac function and the duration of treatment

Parameters	Correlation coefficient (Spearman)	*P* value
LVEF	0.31	0.239
SV	0.169	0.531
LVFS	0.34	0.19
LVMI	-0.049	0.857
Cardiac output	0.48	0.061
Cardiac index	−0.09	0.739
E/A ratio	−0.34	0.188
E wave velocity	−0.42	0.098
A wave velocity	0.11	0.68
Mitral deceleration time	**0.51**	**0.04**
LAD	0.44	0.086
TAPSE	**0.72**	**0.002**
Systolic pulmonary pressure	−0.45	0.075
Aortic diameter	0.37	0.159

*LEVF* Left Ventricular Ejection Fraction, *SV* Stroke Volume, *LVFS* Left Ventricular Fractional Shortening, *LVMI* Left Ventricular Mass Index

The bold texts represent the result with a statistically significant differences

## Discussion

Our study focused on the evaluation of cardiac function among Cameroonian children with congenital adrenal hyperplasia. The aim was to evaluate through standard echocardiography the most common settings for heart function and compared it to a group of healthy children matched for age and sex. With a sample of 57 children (1 case for 2 controls), we found an impaired diastolic function in cases compared to controls and increased values of systolic function in cases. Right cardiac function was similar in the 2 groups.

This pilot study was monocentric but included all patients diagnosed and followed in the country. The lack of references curves of echocardiographic values in the literature for black children [12, 13] makes the interpretation of the values found difficult. The children of this study were not matched according to the fitness level and body composition including the percentage of fat mass, whose importance in cardiovascular health is not negligible [11, 19]. We used the anthropometric parameters and did not take into account the body composition which could influence the interpretation of these results. However, given that none of the children in this study had a body mass index >3 z score, they could all be expected to have normal body fat mass. Moreover, the age of cases and controls (6 years on average) places them at a hyperactive period of life and limits the sedentary lifestyle.

Nonetheless the present study brings relevant data among this particular population.

I. **General characteristics**
  Our study population was younger than that of Sowande and al in Nigeria 2009 [20] who included adolescent and young adults (range 5 weeks to 19 years). Sex rearing proportion was 26.31% (5/19) boys and 73.68% (14/19) girls, and after the karyotype, boys represented 5.26% (1/19). These gender assignment errors were found by Rodrigues and al in 20% of cases in Brazil in 2015 [21], in 30% by Sowande and al in Nigeria in 2009 [20]. In Sudan, Abdullah and al in 2011 [22] in a retrospective study over 5 years involving 122 children, had a higher prevalence of female gender after completion of the karyotype and 19% had to change sex with a preference for males. In Western countries where neonatal diagnosis is made, these sex attribution errors are not found in literature.
  Concerning diagnosis circumstances, the most frequent reason for consultation was abnormal genitalia (clitoris hypertrophy). Sowande and al in 2009 in Nigeria, in their retrospective study on disorders of sexual differentiation, had 6 cases attributed to the CAH and all had as a dominant clinical presentation, clitoromegaly [20].
  The average age at diagnosis was greater than that of Rodrigues and al (2 months) [21]. Awareness of medical teams concerning abnormal genitalia is greater in Latin America, explaining the present difference. The diagnosis is still delayed in developing countries because of limited access to para clinical test for all patients.
  On initial clinical presentation, advanced bone age found in all the patients is related to the late age at diagnosis. Garcia and al in Brazil in 2013 [23], had advanced bone age in 71.43% (10 out of 14) in a population diagnosed and treated before age of 4 months. This highlights the fact that even under treatment, risk of excess androgens circulating is still high. This seems to be controlled in 3 daily doses of hydrocortisone [24].
  The most found type of enzyme deficiency was 11β-OH in 78.9%, which is in contradiction to literature where the deficit in 21-OH (21 Hydroxylase) is recognized as the most common, with a prevalence more than 90% [25, 26]. Indeed in absence of neonatal screening, many children with 21-OH deficiency died during the neonatal period. This also explains the low number of boys in our study population.
  A child in our study had dilated cardiomyopathy diagnosed at 1 year and a clinical improvement was observed upon initial hormone replacement therapy. A similar case was described in Kuwait. Al Jarallah and al in 2004 [27], published a case of rapidly reversible dilated cardiomyopathy. The congestive heart failure and dilated ventricles declined with the introduction of hydrocortisone.
II. **Blood pressure and Cardiac function**
  Blood pressure was higher in cases than controls, either systolic or diastolic. Various studies found similar results: Rodrigues and al [21] and Subbarayan and al [28], found a systolic blood pressure higher among cases.
  Reasons for increase in cardiovascular risk of CAH patients are not totally elucidated. Could high levels of androgens have a vasoconstrictor effect? [29–31]. Moreover, the deficit type involved (11-β OH) has a mineralocorticoid effect by the accumulation of 11-deoxycorticosterone [25, 32]. The likely involvement of treatment with glucocorticoids, resulting in a compromise between the advantages and disadvantages [21]. In 2014, AMR and al in Egypt have shown a rise in cardiovascular risk in CAH patients, but no correlation was found between the different variables and the average daily dose of hydrocortisone. The hypothesis of a cumulative effect of steroid treatment over the years was issued [21, 33]. But our study population is young and treated with doses according to recommendations [1, 25].
  1. **Systolic function of the left ventricle**
    The variables studied to assess left ventricular systolic function were all within normal values for average, but higher in cases than in controls. Although the differences were not statistically significant. The small differences in means between controls and patients could be potentially due to differences in body composition or fitness level. Metwalley and al in Egypt in 2015 [10] studied the left ventricular dysfunction and subclinical atherosclerosis in children and adolescents with classic CAH. He did not found difference between the systolic function of the cases and the controls. However the LVEF of 70.5 ± 3.6 and the LVFS% of 39.2 ± 2.1 were higher than those of controls as in our study.
    LVEF expresses the degree of emptying of the left ventricle. It quantifies the capacity of it and arteries to maintain adequate cardiac output under changing load conditions and / or contractility [34]. Its prognosis value comes from the functional reserves and adaptability to hemodynamic conditions it expresses. This increase in systolic left ventricular activity found among cases may reflect a myocardial stimulation by androgens [3].
  2. **Diastolic function of the left ventricle**
    The E wave velocity and E / A ratio were higher in controls than in cases. In contrast the A wave velocity was higher in cases than in controls. There was no statistically significant difference. The cases deceleration time varied between 78 and 228 ms, which is higher than the controls. This reflects an impaired relaxation function of the left ventricle of cases compared to controls. Metwalley in Egypt, found similar results [10]: an E / A ratio lowered and an extension of time of mitral deceleration and iso volumetric relaxation, indicating left ventricular dysfunction with very significant values *p* < 0.001. In our study this difference was not statistically significant probably because our children were younger. This situation resulted from longer exposure to androgens whose stimulatory activity on the heart was seen with the rise of systolic settings, an abnormal relaxation of the most sought muscle [3, 35].
  3. **Right ventricular function**
    In our study it was assessed mainly by systolic excursion of the plan of the tricuspid annulus. No differences were found between cases and controls for TAPSE. Systolic pulmonary pressure was statistically higher in cases than controls with a mean of 19.86 ± 3.44 mm. Alteration of right ventricular function is usually secondary to left heart failure. However, it can be isolated when it is due to certain lung diseases, right heart or pericardial diseases. Assessment of right ventricular function is, in terms of daily practice, extrapolated over the measures taken left [36]. The echocardiography of the right ventricle faces several difficulties, the main ones being represented by its size and its position in the thorax and especially by its special geometry shaped distorted prism [36]. This explains the difficulties of studying right ventricular function in daily practice and a lack of work on this subject allowing us to compare our results with those of other authors [12].
  4. **Cardiac cavities**
    The left ventricle mass index of the cases was statistically higher than controls. This reflects left ventricular hypertrophy which could be explained by the recognized trophic effect of androgens on cardiomyocytes. All this leading also to an abnormal relaxation of the cardiac muscle [35, 37].
III. ** Correlation between cardiac function parameters and disease determinants**
  A strong correlation was found between the diameter of the left atrium and age at diagnosis. A delayed age at diagnosis, lead to a prolonged exposure to high levels of androgens with their hypertrophic effect on the myocardium [35, 37]. The correlation is even stronger with the diameter of the aorta, coefficient = 0.825 and *p* = 0.00. This could be due to a vasodilator effect at high doses of androgens established in some studies [31].
  Through inhibition of depolarizing calcium influx, it has been established that in humans, their injection at physiological and supra physiological concentrations induces expansion (up to 4.5%) of the coronary artery and improves coronary flow (up 17.5%) in subjects with arteriosclerotic coronary lesions [3].
  A negative significant correlation would have been expected between the duration of the treatment and the cardiac mass, but it is not the case in our study. This highlights the importance of treatment compliance and patients being controlled, for better assessment of the relationship between treatment duration and cardiac mass.

## Conclusions

This study showed that the cases had a greater systolic function than the controls, consequently increasing contractility. It also showed an increased left ventricular mass among cases leading to an altered relaxation of the left ventricle. These abnormalities were highly correlated to the late age at diagnosis and duration of treatment. This hence the necessity to introduce systematic neonatal screening for CAH to ensure early diagnosis and treatment, along with regular monitoring of heart function in children with CAH.

## Abbreviations

11β-OH: 11 Beta-hydroxylase
21-OH: 21 Hydroxylase
BMI: Body mass index
CAH: Congenital adrenal hyperplasia
LAD: Left atrial diameter
LVDd: Left ventricular diastolic diameter
LVEF: Left ventricular ejection fraction
LVFS: Left ventricular fractional shortening
LVMI: Left ventricular mass index
LVSd: Left ventricular systolic diameter
MCC/CBF: Mother and Child Center of the Chantal Biya Foundation
SV: Stroke volume
TAPSE: Tricuspid annular plane systolic excursion
